# Neuroanatomical Substrates of Rodent Social Behavior: The Medial Prefrontal Cortex and Its Projection Patterns

**DOI:** 10.3389/fncir.2017.00041

**Published:** 2017-06-13

**Authors:** Jaewon Ko

**Affiliations:** Department of Brain and Cognitive Sciences, Daegu Gyeongbuk Institute of Science and Technology (DGIST)Daegu, South Korea

**Keywords:** prefrontal cortex, neural circuits, rodent, social behavior, synapse

## Abstract

Social behavior encompasses a number of distinctive and complex constructs that form the core elements of human imitative culture, mainly represented as either affiliative or antagonistic interactions with conspecifics. Traditionally considered in the realm of psychology, social behavior research has benefited from recent advancements in neuroscience that have accelerated identification of the neural systems, circuits, causative genes and molecular mechanisms that underlie distinct social cognitive traits. In this review article, I summarize recent findings regarding the neuroanatomical substrates of key social behaviors, focusing on results from experiments conducted in rodent models. In particular, I will review the role of the medial prefrontal cortex (mPFC) and downstream subcortical structures in controlling social behavior, and discuss pertinent future research perspectives.

## Introduction

In the past three decades, rapid advancements in molecular, cellular and genetic methodologies as well as the implementation of cutting-edge imaging technologies, have accelerated our understanding of social behavior (Insel and Fernald, 2004; Insel, 2010; Stanley and Adolphs, 2013; Gunaydin et al., 2014; Lerner et al., 2016). These new techniques have enabled researchers to explore the neural mechanisms of social bonding, social reward, social aggression, dominance, communication and social network organization. Social behaviors are essential for species survival, which requires the recognition of social interactions in appropriate contexts and the reshaping of individual phenotypes in accordance with various social environments. Yet, the inherent limitations of studying unobservable mind states have limited our understanding of how social perception occurs leads to physiological, cellular and molecular changes associated with social behaviors. The identification of reliable behavioral/physiological correlates in appropriate animal models using a comparative phylogenetic approach and advanced large-scale “-omics” technologies is essential for identifying the evolutionary trajectory of species-unique and species-shared behavioral characteristics (Robinson et al., 2008). Moreover, methodological constraints and intractability issues associated with human studies have driven a need for the development of rodent and non-human primate models of homologous behavioral features. For this reason, researchers have separated behavioral features into simpler, more easily studied elements of social cognition, a strategy that remains highly controversial in comparative psychology. Nevertheless, researchers such as Niko Tinbergen (Bateson and Laland, 2013) have applied this framework to inform elements of social cognition at multiple evolutionary levels, probing the relationships among observable behaviors and determining whether they emerge at analogous stages of brain development, have phylogenetic continuity and serve homologous adaptive functions between species. As a result, the key neural mechanisms underlying social behavior in animal models have become grounded in the fields of biology and biomedicine (Cacioppo and Decety, 2011; Matusall, 2013).

Rodents and non-human primates (e.g., chimpanzees), have served as good models for identifying the neural substrates of social behavior (Cacioppo, 2002; Insel and Fernald, 2004; Crawley et al., 2007; Silverman et al., 2010). A number of studies have shown that rats exhibit a subset of pro-social behaviors that are commonly regarded as part of the behavioral domain of primates, including engagement in reciprocal interactions with conspecifics (Ben-Ami Bartal et al., 2014) and response to the distress of a restrained conspecific by working to release it (Ben-Ami Bartal et al., 2011). Mice also exhibit distinct social behaviors such as territorial aggression and mating through the transmission and interpretation of encountered olfactory signatures as social information (Rennie et al., 2013). Moreover, both rats and mice display emotional contagion, empathic responses, and observational learning (Jeon et al., 2010; Atsak et al., 2011). The expression of these social behaviors depends on the environmental context (e.g., the availability of food; Hurst et al., 1997; Shanahan and Hofer, 2005). In contrast, the prairie vole has been used to study monogamous behavior and pair bonding (Young et al., 1999; Young and Wang, 2004). Anatomical, pharmacological and behavioral analyses in this model have revealed roles for various neurotransmitters and peptide substances in select social behaviors (Numan and Young, 2016). Lastly, optogenetics, predominantly applied in rodents and recently extended to non-human primates, offers an unprecedented opportunity for elucidating the neural substrates of various social behaviors (Yizhar, 2012; Gunaydin et al., 2014).

Given the diversity and complexity of social behavior, it is not unreasonable to expect that conserved neural mechanisms operate across various social species (Skuse and Gallagher, 2011). Moreover, social signals may induce conserved patterns of change in genomic expression through epigenetic modifications, defined as changes in gene expression that are not attributable to changes in DNA sequence (Weaver et al., 2004; Robinson et al., 2008). It is also thought that interactions between genotype and social environment influence the effects of social information on brain function and behavior. Even small variations in early environmental exposure such as exposure to an enriched environment drive striking phenotypic individuality and changes in hippocampal neurogenesis in genetically identical inbred mice (Freund et al., 2013). These intriguing observations highlight the plasticity of social behavior in response to environmental context (Lynch and Kemp, 2014).

Among the vast and complex neural networks involved in social behavior is the PFC and its massive reciprocal connections, which constitute a top-down modulatory system for social behavior (Spencer et al., 2005; Croxson et al., 2011; Bossert et al., 2012; Grossmann, 2013). Reciprocal connections with the PFC involve diverse subcortical structures, including the amygdala for emotional processing, the hypothalamus for stress modulation, the hippocampus for memory processing, the nucleus accumbens (NAc) for social incentive, and regions of the cortex that process sensory and motor inputs and outputs. Ernst and Fudge (2009) proposed a classic “triadic model” that attributes goal-directed motivational behaviors to three functional neural networks that are centered on the PFC, striatum and amygdala, respectively. Accordingly, the goal of this review is to provide an updated perspective on the role of the medial prefrontal cortex (mPFC) and its variable circuit projections in regulating a subset of rodent social behaviors. Of note, it is not the intention of this review to explicitly claim that top-down PFC projections alone constitute the neuroanatomical mechanisms underlying various rodent social behaviors. Rodent behavior is significantly modulated by the dynamics of several other neural systems, including the olfactory system and its connectivity with the posterior amygdaloid and hypothalamic/brainstem circuits (Gross and Canteras, 2012). Yet, given a large body of review literature addressing the roles of other individual neural systems in rodent social behavior, it was my intention to provide an updated overview of the PFC and related subcortical structures in the context of rodent social behaviors.

## The PFC and Related Network Areas Mediating Social Behavior

### PFC

Functional magnetic resonance imaging, electrical stimulation, and lesion studies have identified key brain regions and neural circuits that facilitate social cognition in humans (Martin and Weisberg, 2003; Van Overwalle, 2009). The identified brain regions largely belong to the limbic system and form a complex network of diverse neural circuits related to emotional responses, appetite, sexual behavior, addiction and motivation, and social memory (Tottenham, 2015). Notably, the mPFC has emerged as a crucial neural substrate of social cognition and behaviors in humans (Dolan, 2002; Wise, 2008; Krueger et al., 2009; Grossmann, 2013; Bicks et al., 2015). The human PFC can be anatomically subdivided into the orbitofrontal cortex, dorsolateral PFC, ventrolateral PFC and mPFC, which are collectively involved in complex cognitive behavior and decision-making as well as the moderation of goal-directed social behaviors (e.g., action and outcome monitoring; Amodio and Frith, 2006; Krueger et al., 2009; Yang and Raine, 2009). Not surprisingly, patients with lesions of the mPFC exhibit severe social impairment and reduced behavioral flexibility (Anderson et al., 1999; Drevets, 2000; Eslinger et al., 2004; Forbes and Grafman, 2010).

There is some controversy about the existence of an anatomically comparable PFC structure in rodents (Preuss, 1995; Dalley et al., 2004; Wise, 2008). Yet, emerging evidence suggests that there is significant functional homology between the human and rodent mPFC structures, albeit with clear differences in the level of social cognition supported. Recent studies have also pinpointed divergent functions of anatomically distinct subregions of the rodent PFC (Amodio and Frith, 2006). Therefore, it is reasonable to postulate that comparable brain regions and neural circuits generally contribute to common social behaviors in rodents and humans, albeit in a species-specific manner (Bicks et al., 2015). The translatability of rodent behavioral models is improved by the use of ethologically relevant behavioral paradigms and automated screening platforms for measuring behavior-evoked brain activation (Bicks et al., 2015; Kim Y. et al., 2015).

**Figure 1:**
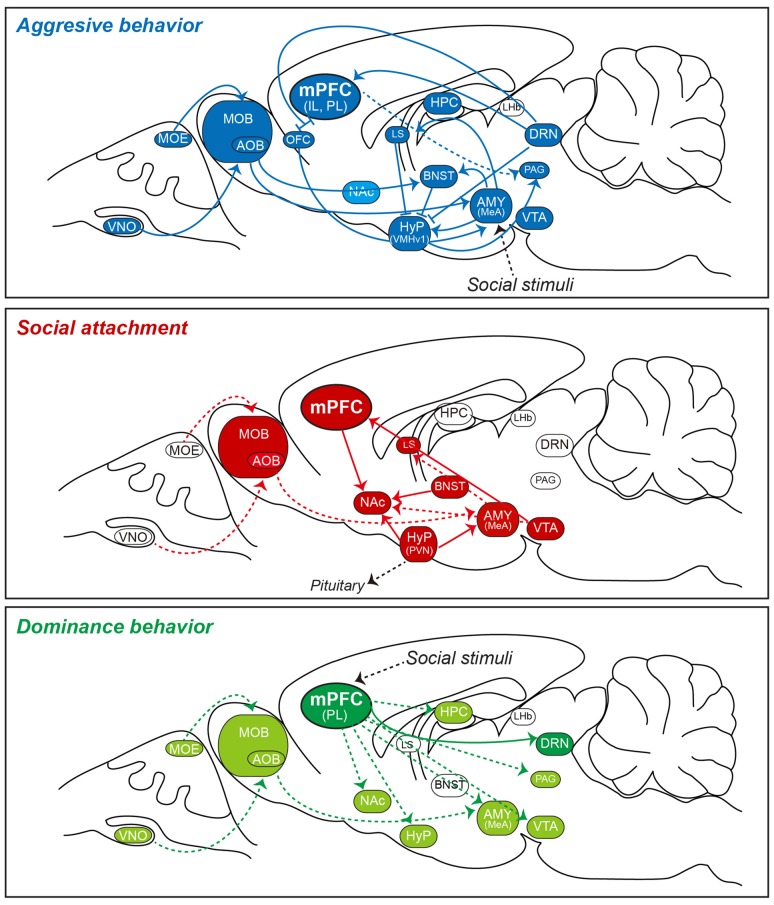
Simplified schematic of social behavior-related neural circuits involving the mPFC in the rodent brain. Shown is a sagittal view of the rodent brain illustrating local and distal circuits implicated in a subset of social behaviors. Recent work using optogenetics, behavioral neuroscience methods, and electrophysiology has established relationships between various social behaviors and activity in specific neural circuits. Note that detailed circuit connectivities between the mPFC and subcortical structures and connections between intra-subcortical structures are not indicated in the figure. Three specific social behaviors (aggressive behavior [top], social attachment [middle], and dominance behavior [bottom]), which are controlled by activation of indicated neural circuits, are presented as exemplaries to highlight that neural circuits underlying aggressive behavior (in blue) and social attachment (in red) are extensively characterized, whereas those underlying dominance behavior (in green) are largely unknown. In rodents, olfactory cues are initially detected by sensory neurons in the MOE and VNO, and are then projected to the MOB and AOB. In turn, these latter regions directly or indirectly transmit information to the MeA. The brain regions that have been implicated in these behaviors (but not clearly verified) are highlighted in either light blue or light green color codes (e.g., NAc for aggressive behavior). Note that there are varying degrees of overlap in the neural circuits involved in these distinct behaviors. It is not known whether the circuitries shown can be generalized to all social animals. Abbreviations: AMY, amygdala; AOB, accessory olfactory bulb; BLA, basolateral amygdala; BNST, bed nucleus of the stria terminalis; CeA, central amygdala; DRN, dorsal raphe nucleus; HPC, hippocampus; Hyp, hypothalamus; IL, infralimbic division of the mPFC; LHb, lateral habenula; LS, lateral septum; MeA, medial amygdala; mPFC; medial prefrontal cortex; MOB, main olfactory bulb; MOE, main olfactory epithelium; MPOA, medial preoptic area; NAc, nucleus accumbens; OFC, orbitofrontal cortex; PAG, periaqueductal gray; PL, prelimbic division of the mPFC; VMHv1, ventrolateral subdivision of the ventromedial hypothalamus; VNO, vomeronasal organ; and VTA, ventral tegmental area.

Top-down PFC projections to subcortical structures, such as the amygdala and hypothalamus, have been proposed to provide executive control and coordinate goal-driven social behaviors in humans (Insel and Fernald, 2004); however, only a few reports have suggested a link between PFC activity and abnormal social behaviors in rodents (Yizhar, 2012; Wang et al., 2014; Figure 1). Moreover, the computational representations by which the mPFC communicates and facilitates the selective coupling of relevant information in downstream subcortical areas have not been systematically investigated in rodents. On a more detailed level, evidence for a causal link between cell type-specific activity and synchronous brain activity in social behavior is generally lacking. Several transgenic mouse lines carrying mutations in genes associated with social neuropsychiatric diseases have been reported to exhibit altered synaptic transmission (Silverman et al., 2010). Yet, molecular, biochemical, and electrophysiological abnormalities in these transgenic mice are quite divergent and not restricted to the PFC alone (Silverman et al., 2010). Therefore, it remains unclear as to whether local alterations in the excitation/inhibition ratio (E/I) and functional desynchronization between different cell types in the mPFC directly causes abnormal social cognition (see Kim et al., 2016 for an alternative perspective). Nevertheless, a partial correlation between mPFC activity and a subset of social behavior-related neuropsychiatric disorders in humans has led to the hypothesis that E/I balance in mPFC circuits may be critical for normal social behavior (Yizhar, 2012; Bicks et al., 2015). For example, using a three-chamber behavioral paradigm that assesses social cognition in the form of general sociability and preference for social novelty, researchers showed that a subset of mPFC neurons exhibited elevated discharge rates while mice approached an unfamiliar mouse, but not when mice approached an inanimate object or empty chamber (Kaidanovich-Beilin et al., 2011); these observations are consistent with the idea that neural activity in the mPFC correlates with social-approach behavior in mice (Lee et al., 2016). mPFC neurons have also been reported to exhibit functional asymmetry between hemispheres in mice, such that the right mPFC was reported to control the acquisition of stress during hazardous experiences while the left mPFC was found to play a dominant role in translating stress into social behavior (Lee et al., 2016). Additionally, knockdown of phospholipase C-β1 in the mPFC impairs social interactions, whereas chronic deletion of the NR1 subunit of the N-methyl-D-aspartate (NMDA) receptor in the mPFC increases social approach behavior without affecting social novelty preference in mice (Finlay et al., 2015; Kim S. W. et al., 2015). NMDA-NR1 dysfunction in the CA3 region of the hippocampus is also sufficient to impair social approach, suggesting that social interaction can be differentially modulated by distinct alterations in a relevant circuit (Finlay et al., 2015). Yet, the positive correlation between mPFC activity and social behavior is not robust to different manipulations of mPFC activity in rodents. Neuroligin-2 is an inhibitory synapse-specific cell-adhesion molecule that was recently implicated in synaptic inhibition in the mPFC. Conditional deletion of the *Nlgn*2 gene in mice produced a gradual deterioration in inhibitory synapse structure and transmission, suggesting that neuroligin-2 is essential for the long-term maintenance and reconfiguration of inhibitory synapses in the mPFC (Liang et al., 2015). Moreover, neuroligin-2-knockout (KO) mice exhibit behavioral abnormalities that are partially correlated with electrophysiological phenotypes at 6–7 weeks but not at 2–3 weeks after gene inactivation (Liang et al., 2015). As a possible explanation for this observation, the authors hypothesized that the behavioral phenotype was produced by dysfunction of a peculiarly plastic subpopulation of inhibitory synapses in neuroligin-2-KO mice (Liang et al., 2015). These studies illustrate the idea that various synaptic signaling and adhesion pathways operating in the mPFC contribute to the initiation, maintenance, and/or modulation of social behaviors. A general goal of future studies should be to establish how common social behavioral impairments in various transgenic mice are related on molecular and synaptic levels. In particular, the optogenetic manipulation of mPFC neurons using the recently engineered Stabilized Step-Function Opsins can help to identify the circuit and synaptic mechanisms that underpin mPFC interactions with specific, distant subcortical regions to regulate various social behaviors (Yizhar et al., 2011; Riga et al., 2014; Ferenczi et al., 2016).

### Amygdala

Recent studies have used cell type-specific optogenetic manipulations in tandem with behavioral testing and electrophysiological recordings to probe the functional coupling of the mPFC and its subcortical systems in the context of social behavior (Yizhar, 2012; Ferenczi et al., 2016). Of the various subcortical networks communicating with the mPFC, the amygdala has been consistently reported to exert modulatory effects over social behaviors by processing emotionally and socially relevant information (Newman, 1999); in rodents, this information is normally sensed through olfactory-based perception (Adolphs, 2010). Accordingly, it has been reported that the size and intrinsic connectivity of the amygdala are highly correlated with social network complexity (Bickart et al., 2011, 2012). Moreover, amygdala lesions lead to alterations in social behavior in rodents (Amaral et al., 2003; Machado and Bachevalier, 2006; Adolphs, 2010; Bliss-Moreau et al., 2013).

The amygdala is composed of more than 20 distinct subnuclei, with one subset serving discrete social behaviors through extensive anatomical and functional connections with other brain areas (McDonald, 1998; Sah et al., 2003; Pape and Pare, 2010; Allsop et al., 2014). The amygdaloid subnuclei are commonly divided into three groups: the basolateral complex (BLA) group that includes the lateral nucleus, basal nucleus, and accessory basal nucleus; a more superficial group that includes the cortical nuclei and nucleus of the lateral olfactory tract; and the centromedial group composed of the medial (MeA) and central (CeA) nuclei (Sah et al., 2003; Marek et al., 2013). The CeA can be further classified into medial and lateral subnuclei (Sah et al., 2003). The BLA is the site of primary sensory input in the amygdala, whereas the CeA is the primary output structure and elicits various physiological fear and social behavioral responses (Sah et al., 2003). Most of the inputs to the BLA are excitatory glutamatergic inputs (Janak and Tye, 2015). Additionally, principal afferent input neurons are locally interconnected via inhibitory interneurons that use γ-aminobutyric acid (GABA) as a neurotransmitter to form feed-back, feed-forward, and lateral inhibitory circuits, depending on the innervation patterns and identities of the involved neurons (Allsop et al., 2014; Ko et al., 2015). In contrast, projection neurons in the CeA are primarily GABAergic, with the central lateral amygdaloid subnucleus projecting to the central medial amygdaloid subnucleus as a main output nucleus of the amygdala (Pape and Pare, 2010). Thus, the excitation of neurons in the CeA leads to the inhibition of target neurons, whereas the inhibition of projection neurons results in increased output from target neurons. The MeA receives strong olfactory inputs and conveys olfactory information to various parts of the hypothalamus to influence various social behaviors, including aggressive, defensive, mating, and parenting behaviors (Canteras et al., 1992, 1995, 2015; Price, 2003; Sokolowski and Corbin, 2012; Nieh et al., 2013; Stuber and Wise, 2016). The lateral nucleus of the amygdala also receives visual, auditory and somatosensory inputs that are conveyed through the CeA, which is preferentially connected to the hypothalamus, brainstem, and mPFC, to govern innate social behaviors like aggression and mating in rodents (Choi et al., 2005; Anderson, 2012; Allsop et al., 2014; Sabihi et al., 2014a,b).

Recent technological advances, including projection-specific optogenetics and other imaging innovations, have permitted researchers to dissect the role of reciprocal connections between the amygdala and mPFC in fear learning, extinction and anxiety-related behaviors (Herry et al., 2008; Tye and Deisseroth, 2012; Marek et al., 2013; Tottenham, 2015; Figure 1). Decreased activity in the mPFC has traditionally been associated with fear generalization in rodents and humans; theta range (4–12 Hz) oscillatory behavior and synchrony in the mPFC-BLA circuit have been linked to the discrimination of aversive vs. safe cues in fear-conditioning and open-field test paradigms in mice (Likhtik et al., 2014). Yet, the interaction of these neural systems during social behavior remains largely uncharacterized. It may not be an overstatement to suggest that similar neural mechanisms in the mPFC-BLA circuit also modulate social behaviors, given the strong comorbidity of anxiety disorders with social cognitive disorders (Peters et al., 2009). The projection patterns of mPFC inputs to the BLA and the functional consequences of these different patterns for distinct phases of social behavior should be delineated. Notably, different MeA neuronal types are responsible for the modulation of social interaction as well as self-grooming in rodents (Hong et al., 2014). Since the amygdala releases a variety of neurotransmitters (e.g., dopamine and serotonin) as well as neuropeptides (e.g., oxytocin [OXT], arginine vasopressin [AVP], corticotropin-releasing hormone [CRH], and neuropeptide Y) that are linked to social behavior, a better understanding these different neurotransmission systems in the mPFC-BLA circuit is a research priority.

### Hypothalamus

The cerebral cortex has efferent output projections to the hypothalamus that affect a wide variety of cardiovascular, gastric and respiratory systems, forming the visceromotor system (Cechetto and Chen, 1990; Ongür et al., 1998). The hypothalamus includes anatomically distinct nuclei that span the periventricular zone (surrounding the third ventricle), medial zone, and lateral zone (Ongür and Price, 2000). The periventricular zone contains neurons that are primarily involved in neuroendocrine and autonomic regulation, whereas the medial and lateral zones influence the somatic motor systems that control motivated behaviors. In rodents, the hypothalamus receives olfactory inputs from the amygdala and other olfactory areas, somatosensory inputs (tactile and pain) from the brainstem, and multimodal sensory inputs from the PFC and hippocampus (Goodson, 2005; Goodson and Kingsbury, 2013; Figure 1). Hypothalamic neurons coordinate a variety of complex homeostatic mechanisms and social behaviors in response to these inputs as well as hormonal input (i.e., OXT and AVP) from the posterior pituitary (Ervin et al., 2015).

Social behaviors influenced by the hypothalamus include reproductive behaviors, aggressive and defensive behaviors, arousal and social affiliation (Choleris et al., 2004; Shelley et al., 2006). Studies in estrogen receptor α subunit-KO mice revealed that the α subunit is critical for lordosis behavior (a simple reproductive behavior) and aggression (Ogawa et al., 1996, 2000). Social isolation stress during adolescence profoundly affects α subunit expression and sexual behavior in female mice (Kercmar et al., 2014). Furthermore, estrogen receptor-β estrogens control OXT production in the paraventricular nucleus (PVN) of the hypothalamus (Choleris et al., 2003), suggesting that the interplay of estrogens and OXT is important for social recognition. Recent studies have shown that optogenetic stimulation of neurons in the ventromedial hypothalamus, which was previously shown to inhibit mating in mice, evokes aggressive behavior towards an inanimate object; this finding highlights the ventromedial hypothalamus as a possible neural substrate for competitive interactions between fighting and mating (Pfaff and Sakuma, 1979; Lin et al., 2011). It is also known that tyrosine hydroxylase-expressing neurons in the anteroventral periventricular nucleus of the hypothalamus govern instinctive parental behaviors in a sex-specific manner by regulating circulating concentrations of OXT (Scott et al., 2015). Yet, whether neurons in distinct hypothalamic nuclei affect specific social behaviors, particularly those regulated by upstream inputs from the amygdala, hippocampus and cerebral cortex, remains unknown.

An organism’s internal state modifies the response patterns of the hypothalamus to particular stimuli. Consummatory behavioral responses are mediated by projections to lower brainstem neurons, such as those in the periaqueductal gray (PAG), which then regulate the output of cranial and spinal motor neurons. Alternatively, the hypothalamus connects to dopaminergic neurons of the ventral tegmental area (VTA) and amygdala, which play primary roles in regulating goal-directed behavior (Figure 1). Furthermore, the PFC modulates output from the amygdala and ventral striatum to facilitate an adaptive balance between the two systems. In consideration of these observations, it can be hypothesized that separate and distinct neuronal populations within the hypothalamus differentially monitor and respond to an organism’s internal state and social context, and relay this information to the PFC for the orchestration of motivational processes (Ernst and Fudge, 2009).

### PAG

The PAG is a critical portion of the limbic midbrain that integrates autonomic, behavioral and anti-nociceptive stress and fear responses (Behbehani, 1995). The PAG is organized into separate columns with distinct anatomical, physiological, pharmacological and functional features (Keay and Bandler, 2002; Millan, 2003). Within the cardiovascular-controlling network, the PAG is divided along its rostrocaudal axis into dorsomedial, dorsolateral, lateral, and ventrolateral columns (Carrive, 1993). Notably, the limbic-hypothalamic-midbrain PAG axis has been proposed as a principal neuroanatomical substrate that regulates various forms of aggressive behavior, particularly in felines, which have been used as a standard animal model for studying the neural mechanisms of aggression and rage (Zalcman and Siegel, 2006). Electrical or chemical stimulation of the dorsomedial and dorsolateral PAG produces defensive/rage-like and predatory-attack behaviors, emotional and motivational processing, and vocalization in feline (Graeff, 1994; Behbehani, 1995; Zalcman and Siegel, 2006). Interestingly, the dorsal PAG interacts with the amygdala and medial pre-optic area to partially regulate active defensive behavior in response to innate danger stimuli, whereas the ventral PAG is associated with passive defensive behavior (freezing) in response to conditioned danger stimuli in rodents (Brandão et al., 2008). Consistent with this idea, dysfunction of the dorsal PAG has been implicated in human panic disorder (Schenberg et al., 2001).

The PAG is rich in excitatory glutamate receptors and has high densities of both presynaptic and postsynaptic excitatory amino acid receptors. Cross-species comparative studies have shown considerable similarities in neuronal types and distributions within the PAG, including: fusiform neurons, multipolar neurons, stellate neurons, pyramidal neurons and ependymal neurons (Behbehani, 1995). Defensive behavior induced by the activation of excitatory amino acid receptors in the dorsal PAG is modulated by serotonin (Beckett and Marsden, 1997). Consistent with this idea, pharmacological elevation of serotonin levels in the PAG produces anti-aversive effects (Deakin and Graeff, 1991; Lovick et al., 2000). In particular, NMDA receptors expressed in PAG neurons interact with glycine-B receptors and 5-hydroxytryptamine (5-HT) receptors to mediate defensive and anxiety-related behaviors in rats, respectively (Carobrez et al., 2001; Moraes et al., 2008). Different subtypes of 5-HT receptors activate distinct neural pathways in the dorsal PAG to influence the net activity of output neurons; in turn, these output neurons are regulated by complex interactions between excitatory on-cells and inhibitory off-cells in the PAG (Brandão et al., 2008). Among 5-HT receptors, 5-HT_2_ receptor activation provides excitatory input to off-cells, whereas 5-HT_1A_ receptor activation mediates inhibitory input to on-cells. These observations are in agreement with the idea that the major intrinsic circuit within the PAG is a tonically active GABAergic network, and that inhibition of this circuit constitutes an important mechanism for producing PAG output (Brandão et al., 2008).

The neuropeptides substance P and cholecystokinin facilitate defensive rage behavior through neurokinin-1 and cholecystokinin-B receptors, respectively (Zalcman and Siegel, 2006). In contrast, defensive behavior is suppressed by the activation of μ-opioid receptors on PAG neurons via enkephalinergic input from the CeA (Zalcman and Siegel, 2006). Cytokines such as interleukin-1β and interleukin-2 also modulate defensive rage behavior through interactions with various neurotransmitter mechanisms (Zalcman and Siegel, 2006).

Although tracer studies in various animal models have demonstrated that threat-coping behavior is initiated by mPFC input to the PAG (Floyd et al., 2000; Keay and Bandler, 2001; Gabbott et al., 2005; Franklin et al., 2017) except in the circumstance of fear and anxiety initiated by PAG-amygdala circuitry (Johansen et al., 2010; McNally et al., 2011; Kim et al., 2013; Penzo et al., 2014), the physiological roles of specific neural circuits that interconnect PAG neurons with other brain networks are largely undefined. Future studies are required to elucidate the specific roles of PAG circuits for processing of fear as an important trigger for aggression, hierarchy, social fear and social communication (Adolphs, 2013).

### Dorsal Raphe Nuclei

As indicated above, 5-HT serves a variety of cognitive functions, including motivational behavior, attention, stress coping, value-based decision making (reward), social behavior and learning and memory (Nakamura, 2013). 5-HT is primarily synthesized and released from the dorsal raphe nucleus (DRN), a heterogeneous nucleus in the brainstem that provides major serotonergic afferents to the forebrain, including the ventromedial PFC (Michelsen et al., 2008; Challis and Berton, 2015). Strong evidence suggests that the ventromedial PFC is connected to the DRN via reciprocal monosynaptic projections; recent work using advanced tract-tracing methods and transgenic mice in combination with ultrastructural methods and *in vivo* electrophysiology have accelerated the characterization of this pathway (Gonçalves et al., 2009; Vazquez-Borsetti et al., 2009; Challis and Berton, 2015; Figure 1). Electron microscopy of mPFC afferents in contact with the DRN and electrophysiological analyses using GABA antagonists indicate that mPFC terminals preferentially synapse with GABA-labeled dendrites and not dendrites labeled with the 5-HT marker tryptophan hydroxylase 2 (Celada et al., 2001; Jankowski and Sesack, 2004). A more recent study using transgenic mice with fluorescently labeled GABAergic or 5-HT neurons (Challis et al., 2013) demonstrated little overlap between the regions occupied by GABA and 5-HT in the DRN. Additionally, GABA and 5-HT neurons differ in size and intrinsic excitability (Shikanai et al., 2012; Gocho et al., 2013). Optogenetic approaches have demonstrated that DRN GABAergic neurons inhibit 5-HT neurons (Challis et al., 2013). Moreover, ventromedial PFC axons form synapses with both GABAergic- and 5-HT neurons in a topographically distinct manner (Challis et al., 2014). Using an anterograde viral vector system that selectively expresses a fluorescent synaptophysin-GFP fusion protein in pyramidal neurons of the ventromedial PFC and channelrhodopsin-2–assisted circuit mapping technology (Petreanu et al., 2007), Challis et al. (2014) showed that mPFC neurons form synapses with rostral and caudal DRN neurons where there are clusters of GABAergic neurons. These data suggest that DRN GABAergic neurons are in a critical position to receive top-down regulatory input from the ventromedial PFC, but do not provide functional evidence for connectivity between the ventromedial PFC and DRN. Two recent studies have challenged this hypothesis: using a modified rabies virus system to retrogradely map synaptic connections between cells in the PFC and DRN, it was found that PFC inputs amounted to roughly 15% of the total synaptic input received by DRN neurons (~10% of input onto 5-HT neurons and ~5% onto GABAergic neurons), suggesting that cortical inputs to the DRN likely have weaker functional influence compared to other subcortical inputs (e.g., to the hypothalamus; ~30% of input; Pollak Dorocic et al., 2014; Weissbourd et al., 2014). Inconsistencies among these studies might be attributable to slightly different experimental systems and technical factors, such as the use of different adeno-associated virus serotypes, virus target areas, and/or mouse transgenic strategies.

The mPFC-DRN projection has relevance to depressive-like states in rodents (Hamani et al., 2012; Albert et al., 2014; Mahar et al., 2014; Riga et al., 2014; Veerakumar et al., 2014). Intriguingly, optogenetic activation of the mPFC-DRN excitatory pathway produced opposing behavioral effects in two different assays of depression-like behavior in rodents: whereas Warden et al. (2012) showed that activation of the mPFC-DRN circuit promoted pro-social behavior in the forced-swim test, Challis et al. (2014) demonstrated that activation produced avoidance of a social target in the chronic social defeat paradigm. Although the reasons for contradictory results in these studies are not entirely clear, it is possible that the mPFC-DRN pathway differentially regulates social interaction and despair behaviors. Alternatively, differential effects of acute and chronic photoactivation of the mPFC-DRN pathway or potential off-target effects of optogenetic manipulation might account for these conflicting observations (Otchy et al., 2015). Nonetheless, these experiments clearly demonstrate the involvement of specific mPFC-DRN projections in adaption during socioaffective behaviors.

It was recently reported that dopaminergic neurons in the DRN play a role in social exclusion or feelings of social disconnection (i.e., loneliness-like states) in rodents (Robinson and Ben-Shahar, 2002; Gunaydin et al., 2014; Matthews et al., 2016). DRN neuron function and plasticity are sensitive to acute social isolation, such that they are considered to be a key neural substrate of the social monitoring system (Gardner et al., 2005). Additionally, DRN neurons elicit the release of both dopamine and glutamate in downstream structures, eliciting increased calcium signals upon initial social contact following social isolation and forming dense projections to distinct subnuclei of the amygdala (Matthews et al., 2016). Strikingly, optogenetic activation of dopaminergic DRN neurons was found to promote social preference in group-housed mice, whereas the same manipulation in the absence of a social target produced an aversive state, suggesting that the activity of these neurons represents the subjective experience of social isolation (Matthews et al., 2016). Although the roles of neural circuits involving DRN dopamine neurons have been partly characterized, additional work is required to understand the significance of connectivity between DRN neurons and several other brain regions including the mPFC. Dissecting the coordinated interplay among dopamine, glutamate and other neuromodulators should prove fruitful in providing a more complete understanding of how various neurons in the DRN interact with upstream and downstream areas to modulate distinct social behaviors.

### CA2 Subfield of the Hippocampus

It is not surprising that mPFC neurons form major connections with the hippocampus to mediate social cognition in rodents, given the critical role of the hippocampus in memory formation (Kogan et al., 2000; Bicks et al., 2015; Finlay et al., 2015; Figure 1). A number of studies suggest that activity in a distinct frequency range is correlated or synchronized between the mPFC and hippocampus during social cognition (Churchwell and Kesner, 2011; Euston et al., 2012; Bicks et al., 2015). In particular, the ventral hippocampus (vHPC) has been implicated in emotional behaviors, such as fear and anxiety (Bannerman et al., 2004a; Tovote et al., 2015) as well as social memory (Okuyama et al., 2016). Consistent with these observations, postnatal inactivation of the NMDA receptor NR1 subunit or injection of an NMDA receptor antagonist induces a social withdrawal phenotype in mice (Corbett et al., 1995; Sams-Dodd, 1996; Gandal et al., 2012). Moreover, deletion of the NR1 subunit in GABAergic interneurons of the cortex and hippocampus blunts short-term social memory without affecting social interaction behaviors (Belforte et al., 2010; Jeevakumar et al., 2015; Jeevakumar and Kroener, 2016). A recent study examined the different effects of NMDA receptor dysfunction in the mPFC vs. the CA3 region of the hippocampus with regard to social approach and social novelty preference and found that receptor dysfunction in the dorsal CA3 region impaired social approach but not social novelty preference, whereas excitotoxic lesions of the mPFC increased social interaction (Avale et al., 2011; Finlay et al., 2015). These data suggest that the localized dysfunction of NMDA receptors in the mPFC and hippocampus differentially affect social behavior.

The vHPC also has robust and reciprocal connections with the amygdala that are important for the expression of social behaviors in rodents (Cadogan et al., 1994; Deacon et al., 2002; Kjelstrup et al., 2002; Bannerman et al., 2004b; McHugh et al., 2004; Kheirbek et al., 2013). Specific projections from a given brain region can encode information that cannot be conveyed by the generalized activation or inhibition of an entire brain region (Tye et al., 2011). Indeed, a majority of BLA neurons mediate anxiogenic effects via projections to regions implicated in anxiety such as the mPFC, the bed nucleus of the stria terminals, and the vHPC; whereas the BLA-central lateral amygdaloid subnucleus circuitry specifically mediates an anxiolytic phenotype (Bishop, 2007; Tye et al., 2011; Calhoon and Tye, 2015). The contribution of specialized connections between the BLA and vHPC to social behavior was recently investigated by expressing channelrhodopsin-2 in glutamatergic BLA projection neurons and illuminating BLA axon terminals within the vHPC; inhibition of the BLA-vHPC circuit increased sociability whereas excitation of this pathway decreased sociability as measured in two different behavioral paradigms (i.e., the three-chamber sociability test and juvenile-intruder test; Felix-Ortiz and Tye, 2014). Thus, glutamatergic transmission in the BLA-vHPC circuit controls both anxiety-like behavior and social interaction, providing a mechanistic link for these behaviors (Tye et al., 2011). Moreover, these studies suggest that, although social behaviors involve a broad neural network distributed across multiple brain regions, social interaction can be modulated by the manipulation of a single key circuit element (File and Seth, 2003; Allsop et al., 2014). A recent study further complicated this interpretation, showing that inhibition of the vHPC impaired social memory, and that a subset of vHPC neurons was more strongly activated in response to a familiar mouse than in response to a non-familiar mouse. Strikingly, optogenetic stimulation of neurons in the relevant vHPC subpopulation restored familiarization memory, suggesting that vHPC neurons and their NAc shell projections are critical components of social memory storage (Okuyama et al., 2016).

In this review article, I have mainly described studies involving the hippocampal CA2 region based on its recently discovered link with social cognition (Chevaleyre and Piskorowski, 2016; Dudek et al., 2016). Although the CA2 region was initially described by Lorente de Nò (1934) as an anatomically distinct structure (Dudek et al., 2016), this region has generally been ignored by neuroscientists and omitted from standard hippocampal circuit diagrams. In addition to extrahippocampal inputs such as those from vasopressinergic neurons in the PVN, medial raphe nucleus neurons, and supramammillary nucleus neurons, CA2 receives bilateral inputs from the CA3 Schaffer collaterals and newborn dentate gyrus granule cells (Borhegyi and Leranth, 1997; Shinohara et al., 2012; Cui et al., 2013; Zhang and Hernandez, 2013; Llorens-Martín et al., 2015). Unlike CA3 pyramidal neurons that innervate the apical dendrites of CA1 pyramidal neurons (the stratum radiatum), major projections from CA2 innervate the basal dendrites of CA1 pyramidal neurons (the stratum oriens; Shinohara et al., 2012; Cui et al., 2013). Extrahippocampal CA2 projections include axons that form reciprocal connections with the supramammillary nucleus, septal nuclei and medial entorhinal cortex (Cui et al., 2013; Rowland et al., 2013); however, the functional significance of these distinct circuitries has not been investigated in detail.

CA2 pyramidal neurons are anatomically and physiologically distinct from those in CA1 or CA3 in that their apical dendrites are branched and optimized for initiating sodium spikes at distal synapses in the stratum lacunosum-moleculare, which are communicated to cell bodies arising from layer II of the entorhinal cortex (Chevaleyre and Siegelbaum, 2010; Piskorowski and Chevaleyre, 2012; Sun et al., 2014). Moreover, CA2 neurons are electrophysiologically characterized by a large capacitance, low input resistance and high resistance to long-term potentiation (Chevaleyre and Siegelbaum, 2010; Caruana et al., 2012; Hitti and Siegelbaum, 2014). The number of interneurons in CA2 is higher than that in CA1 or CA3, suggesting that the local inhibitory circuitry in CA2 represents a powerful brake on pyramidal neuron firing (Piskorowski and Chevaleyre, 2013; Botcher et al., 2014). The unique functional characteristics of CA2 compared to CA1 or CA3 likely stem from differences in anatomical organization, input and output patterns, and gene expression (Lein et al., 2004). Although sets of CA2-enriched genes have been identified, the putative mechanisms underlying the unique features of CA2 are not clearly defined (Dudek et al., 2016). Insight into the specific role of the CA2 area in memory and other hippocampus-dependent behaviors has come from studies using transgenic mice lacking CA2-enriched genes (Lee et al., 2010; Dudek et al., 2016). CA2 neurons are more sensitive to contextual cues, slightly larger and more abundant in place fields, and have higher firing rates than neurons in CA1 or CA3 regions; these properties suggest that CA2 circuits preferentially support temporal rather than spatial aspects of hippocampal-dependent memory (Wintzer et al., 2014; Mankin et al., 2015). One study examined how social stimuli (e.g., exposure to novel or familiar animals) affected the firing rate of CA2 neurons in rats and found that the presentation of social stimuli elicited global remapping of place fields in CA2, but not changes in firing rate or immediate-early gene expression (Alexander et al., 2016). Further evidence indicating a specific role for CA2 in social behavior is that CA2 pyramidal neurons express high levels of the OXT and vasopressin-1b receptors, which are established regulators of social behavior (Young et al., 2006; Stevenson and Caldwell, 2012; Smith et al., 2016). Indeed, targeted optogenetic activation of CA2 vasopressin terminals that originate in the PVN of the hypothalamus enhances social memory in mice during the acquisition phase but not the retrieval phase (Smith et al., 2016). Thus, CA2 appears to facilitate the salience of social signals (Dudek et al., 2016). Future studies using CA2-specific Cre-driver mouse lines should systematically examine whether CA2-enriched genes play important roles in the memory encoding of social information (Hitti and Siegelbaum, 2014). Moreover, the way in which CA2 neurons integrate social processing with other aspects of episodic memory (e.g., time and space) and how CA2 interacts with other brain networks to mediate social behaviors should be investigated.

### Lateral Habenula

The lateral habenula (LHb) is part of the epithalamus that relays emotional/internal state information and influences both the dopamine and 5-HT systems (Lecourtier et al., 2008; Sego et al., 2014). Several reports have suggested that the LHb is involved in olfactory processing, mating behavior, aversive or reward learning, and the execution of complex goal-directed actions (Baker et al., 2015). The LHb is divided into as many as 10 subdivisions based on projection neuron targets or neuronal types, but the specific contributions of each individual subregion is not clear (Geisler et al., 2003; Aizawa et al., 2012; Wagner et al., 2016). The LHb can also be divided into medial and lateral segments; the medial segment mainly projects towards the median and DRN, whereas the lateral segment projects to the rostromedial tegmental nucleus (Proulx et al., 2014). The LHb receives inputs from various brain regions, including the basal ganglia, lateral preoptic area, lateral hypothalamus, VTA, basal forebrain (BF) and medial raphe nuclei, which suggests that it plays a role in behavioral flexibility (Lecourtier and Kelly, 2007; Hikosaka, 2010; Quina et al., 2015). In particular, the medial LHb receives inputs from prelimbic and infralimbic regions of the mPFC, while the lateral LHb receives inputs from the anterior cingulate cortex and insular cortex (Baker et al., 2015). The LHb differentially affects tonic and burst firing aspects of dopaminergic neurotransmission by forming direct excitatory projections onto GABAergic interneurons and indirect projections to the rostromedial tegmental nucleus, ultimately influencing goal-directed behaviors (Lecourtier et al., 2008; Zweifel et al., 2009; Brinschwitz et al., 2010; Balcita-Pedicino et al., 2011; Klanker et al., 2013). The LHb also projects to two main 5-HT nuclei, the DRN and MRN (Vasudeva et al., 2011). In general, prefrontal information about cognitively demanding tasks is processed and integrated with other inputs in the LHb for goal-directed learning (Baker et al., 2015).

A recent study reported a role for the LHb in social play behavior, which is a vigorous form of social interaction in young mammals that facilitates the formation and maintenance of social communication and bonding (Baarendse et al., 2013; van Kerkhof et al., 2013). Interestingly, while the lateral LHb is involved in signaling aversive stimuli via the rostromedial tegmental nucleus-VTA pathway to inhibit the dopaminergic activity (Hong et al., 2011; Lammel et al., 2012; Stamatakis and Stuber, 2012), the medial LHb alters activity in the habenula-VTA feedback loop in response to social play behavior after social isolation (van Kerkhof et al., 2013). Additionally, the LHb has been implicated in regulating serotonin and noradrenaline to modulate social play behavior (Trezza et al., 2010; Siviy and Panksepp, 2011). Signaling from the basal ganglia to the habenula in response to aversive stimuli is attenuated by serotoninergic input, and anti-depressants decrease the activity of the medial segment of the LHb in rodents (Shabel et al., 2012). Thus, the positive experience of social play behavior appears to decrease habenula activity in the context of a negative emotional state, such as after social isolation. The effect of habenula inactivation is specific for social play behavior, as it does not affect social exploration or locomotor activity (Lecourtier et al., 2004; van Kerkhof et al., 2013). Therefore, the habenula appears to process both positive and negative social information in order to produce a correct balance of modulatory neurotransmission and facilitate social play (Trezza et al., 2010; Siviy and Panksepp, 2011).

### Olfactory System

Most of the social behaviors described in the current review are initiated by activation of the olfactory system. Rodents rely heavily on olfactory cues for social interaction; in turn, odor-dependent social learning depends on neuromodulation of the olfactory system (Linster and Fontanini, 2014; Choe et al., 2015). It has been shown that OXT plays a central role in both appetitive and aversive social odor learning by acting on an ensemble of OXT receptor-expressing cells originating in the piriform cortex (Choe et al., 2015). Moreover, the OXT/OXT receptor signaling axis encodes the saliency of social stimuli but not that of non-socially rewarding stimuli by entraining neutral sensory representations to social cues (Choe et al., 2015). A recent study proposed a synaptic mechanism to account for odor-specific social regulation by OXT that invokes the top-down recruitment of GABAergic interneurons (Oettl et al., 2016). It is likely that OXT increases signal-to-noise ratios of target social circuits by improving the temporal precision and fidelity of information transfer and elevating inhibitory tone (Owen et al., 2013; Marlin et al., 2015). OXT activity has also been suggested to modulate sexually dimorphic circuitry to govern parental behavior (Rilling and Young, 2014; Wu et al., 2014), suppress fear responses through connections with the CeA and spinal cord (Knobloch et al., 2012; Eliava et al., 2016), and drive consolation-like behavior and social reward via projections to the NAc (Dölen et al., 2013; Wang et al., 2013; Burkett et al., 2016). Importantly, optogenetic stimulation of tyrosine hydroxylase-positive neurons in the hypothalamus increases the number of monosynaptic inputs to OXT-expressing neurons in the PVN, thereby regulating OXT secretion (Scott et al., 2015). Moreover, social experience modifies OXT-dependent synaptic plasticity by modulating OXT binding to canonical receptors expressed in the DRN (Dölen et al., 2013). Intriguingly, environmental sensory experience regulates cross-modal synaptic plasticity through OXT signaling in sensory cortical neurons (Zheng et al., 2014). Furthermore, OXT receptors expressed in somatostatin-positive and regular-spiking interneurons of the mPFC modulate rodent female social and emotional behavior (Nakajima et al., 2014). Corticotropin-releasing-hormone binding protein (CRHBP), which inhibits the function of the stress hormone CRH, is specifically expressed in OXT receptor-expressing interneurons and specifically blocks CRH-induced potentiation in postsynaptic layer II/III pyramidal neurons in male mice, suggesting a gender-, cell type-, and state-specific role for OTX/OTX receptor signaling and the CRHBP/CRH pathway in the mPFC (Nakajima et al., 2014; Li et al., 2016). These observations generally reinforce the idea that multiple neuromodulators act in concert to trigger social behaviors in synergistic and/or antagonistic manners. Some key questions regarding the role of OXT in social behavior are yet unresolved. For example, it is unclear as to how OXT specifically drives complex social behaviors. To this end, it is not unknown how OXT administration leads to prosocial effects, given the complicated interactions of OXT systems with the blood-brain barrier.

## Future Perspectives

Aided by new interdisciplinary approaches and multi-level analyses for understanding social behavior and cognition, social neuroscience has blossomed into a comprehensive and rapidly advancing field of research (Adolphs, 2009). In particular, sociogenomics has been integrated into various scientific fields, including classical psychology and modern neuroscience, to improve our understanding of the molecular basis of social behaviors. The genetics of social behavior have been rapidly and successfully explored in mice as well as non-human primates, providing a wealth of additional knowledge about social function and disease in recent decades (Robinson et al., 2005). In the current review article, I have attempted to synthesize key experimental observations that are central to extracting the general principles of behavioral circuits in rodents. This focus reflects the important assumption that key organizational features of neural systems are conserved across distantly related species that exhibit diverse forms of social behaviors (Robinson et al., 2005). In this context, it is critical to analyze how specific social behaviors can be divided into simpler components and used to formulate a coherent picture of the universal genetic complement of a social animal. Various synaptic pathways are obvious candidates, and the significance of these pathways for social behaviors and social interaction in particular has been consistently documented using transgenic mouse models of autism spectrum disorder and schizophrenia. Yet, linking synaptic molecular pathways to phenomenological outcomes in animal models has proven problematic in many cases. Although the large gap between genetic and systems approaches remains a challenge, sophisticated tools for mapping the structural and functional neural circuits that mediate social behavior will continue to allow the identification of neural substrates of social behaviors. Two complementary research directions should be pursued to narrow this gap. First, it is critical to understand how the regulated release of various social neuropeptides coordinates distinct neural circuit dynamics. Second, future research should delineate the distinct neural systems involved in social and non-social behaviors in various spatiotemporal contexts of animal behavior.

## Author Contributions

JK wrote the manuscript.

## Conflict of Interest Statement

The author declares that the research was conducted in the absence of any commercial or financial relationships that could be construed as a potential conflict of interest.
